# Project INTEGRATE: Developing a Framework to Guide Design, Implementation and Evaluation of People-centred Integrated Care Processes

**DOI:** 10.5334/ijic.4178

**Published:** 2019-02-01

**Authors:** Lucinda Cash-Gibson, Olena Tigova, Albert Alonso, George Binkley, Magda Rosenmöller

**Affiliations:** 1 Universitat Pompeu Fabra, Ramon Trias Fargas, 25–27, Barcelona, Catalonia, ES; 2 Institut Català d’Oncologia, Avinguda de la Granvia, 199–203, L’Hospitalet de Llobregat, Barcelona, Catalonia, ES; 3 Bellvitge Biomedical Research Institute, Avinguda de la Granvia, 199, L’Hospitalet de Llobregat, Barcelona, Catalonia, ES; 4 Hospital Clínic, Calle Villarroel 170, Barcelona, Catalonia, ES; 5 IESE Business School, University of Navarra, Avenida Pearson 21, Barcelona, ES

**Keywords:** Delivery of Health Care, Integrated Care, People centeredness, Framework, Management

## Abstract

**Background:**

People-centred integrated care is an acknowledged approach to improve the quality and effectiveness of health systems in delivering care around people’s needs and preferences. Nevertheless, more guidance on how to effectively design, implement and evaluate the care process of people-centred integrated care services is needed. Under Project INTEGRATE, a framework was developed to guide managers in the assessment, transformation and delivery of these health service innovations.

**Methods:**

The framework is a product of the synthesis of operations, service and project management literature, relevant health care literature, and the analysis of four good practice integrated care case studies analysed under Project INTEGRATE. A first iteration of the framework was developed and then applied to one of the integrated care case studies to test its validity and utility.

**Results and Discussion:**

The tool combines a number of important considerations and criteria that have not been previously included in integrated care assessment frameworks, allowing for a pragmatic and comprehensive analysis of the care process.

**Conclusion:**

This framework can be used as a stand-alone or combined tool to guide managers to plan and evaluate the care process design of people-centred integrated care services; future work should apply this tool to other settings.

## Introduction

In the context of a growing burden of chronic conditions and multi-morbidity, technological advancements and financial constraints [1], there is an urgent need to transform health systems to better meet current and future demands for care and to improve population health and wellbeing outcomes [2]. A people-centered integrated care (PCIC) approach recognizes these challenges faced by health care systems and aims to transform them and enhance their overall performance and sustainability, by reducing fragmented delivery of services, duplication of efforts and resources and improving service-user satisfaction in line with their health and well-being needs and care preferences [3,4,5,6,7,8]. As such, PCIC has become a central part of policy initiatives to improve the access, quality and effectiveness of health and social care systems [6,9,10].

Many countries have implemented health services reforms to shift away from fragmented provider-centred models of care and reorient them around people and communities to ensure that everybody has equitable access to a continuum of care that is responsive, coordinated and effective, as well as efficient, safe and of quality [5,11]. Whilst the concepts of integrated care and people-centredness (i.e. the “what”) have become widely acknowledged, there is less clarity and consensus on the ‘how to’ implement them in different settings [2,6,12,13,14,15,16]. Previous research has described the need to move towards PCIC for the management of chronic and complex conditions in practice, the core components of PCIC, and the managerial attributes needed to coordinate this process. However, there is limited knowledge of how to successfully implement PCIC [17] and how to transfer successful examples of these experiences and service innovations to other settings [18,19].

Processes connect multiple entities together with an overall purpose. A health care process can be thought of as a service user going through a sequence of activities or a chain of events, over a period of time, with interactions with different staff, services and facilities, as well as with other service users and occasionally with a series of unintended incidents not under the control of the service user or providers [20]. To successfully introduce service innovations such as PCIC, requires a comprehensive understanding of these interconnected activities, components and stakeholders involved, at multiple levels and over time in a given context [21,22], and this therefore raises significant challenges for managers and planners [13,23,24,25,26]. A comprehensive understanding and guidance on how to effectively design, implement, and assess the impact and scale-up of PCIC in different settings is required for managers and planners.

In the Global Framework on Integrated People-Centred Health Services [26] and the World Health Organization (WHO) European Region’s Framework for Action on Integrated Health Services Delivery [9], WHO calls for actions across several domains, one of which is to facilitate the strategic management of health service transformations towards integrated care.

Operations, change and project management strategies, can serve as valuable tools in many sectors, including the health sector, to facilitate the strategic management of service transformations and guide the operationalisation of the ‘how to’ components in process implementation by setting out the tasks to be undertaken to realise the change process and to support its continual improvement [27]. In turn this can then ensure a positive impact on service users (e.g. improved satisfaction with services and improvements in their health outcomes), on the professionals involved in delivering the service (e.g. improved professional satisfaction, retention and development), as well as on service providers (e.g. reduced expenditure and hospital readmissions) [22]. The lean management approach for example, has been successfully adopted in health care settings to improve quality and safety in health care [28,29,30].

One model was developed to assist managers to determine whether the essential integrated care elements are in place at the different implementation phases, so as to provide insights for further improvement [31]. Whilst the tool consolidates integrated care knowledge into a managerial guide, it does have some limitations in that it only indirectly includes service-user perspectives, governance issues are not comprehensively covered, and it is unclear whether it can be used to support complex health and social care needs assessments [21,32].

One aim of Project INTEGRATE, a European Commission FP7 funded collaborative research project (2012–2016) [33], was to gain insights into the leadership, management and delivery of integrated care to support European health systems in their respond to the challenges of ageing populations and the rise of people living with long-term conditions [18,34]. One specific task under the project was to develop a PCIC process framework with a two-fold aim; firstly, to support health care service providers and managers to better monitor and evaluate their established PCIC services and secondly, to support those without established PCIC services to comprehensively plan, implement and evaluate them. In this paper we describe the development of this framework.

## Methods

The framework aims to provide guidance for health care service managers to self-assess their organisation’s integrated health services in particular the care process aspect, to ensure that services are designed and delivered in line with people’s health needs and care preferences. In addition, the framework can be used to ensure best management practice is applied to the process for effective delivery of PCIC, and to assess the impact and added value of such service innovations on the various stakeholders involved at each stage of the PCIC process, irrespective of the current level of maturation of the PCIC service in place or the context.

**Figure 1:**
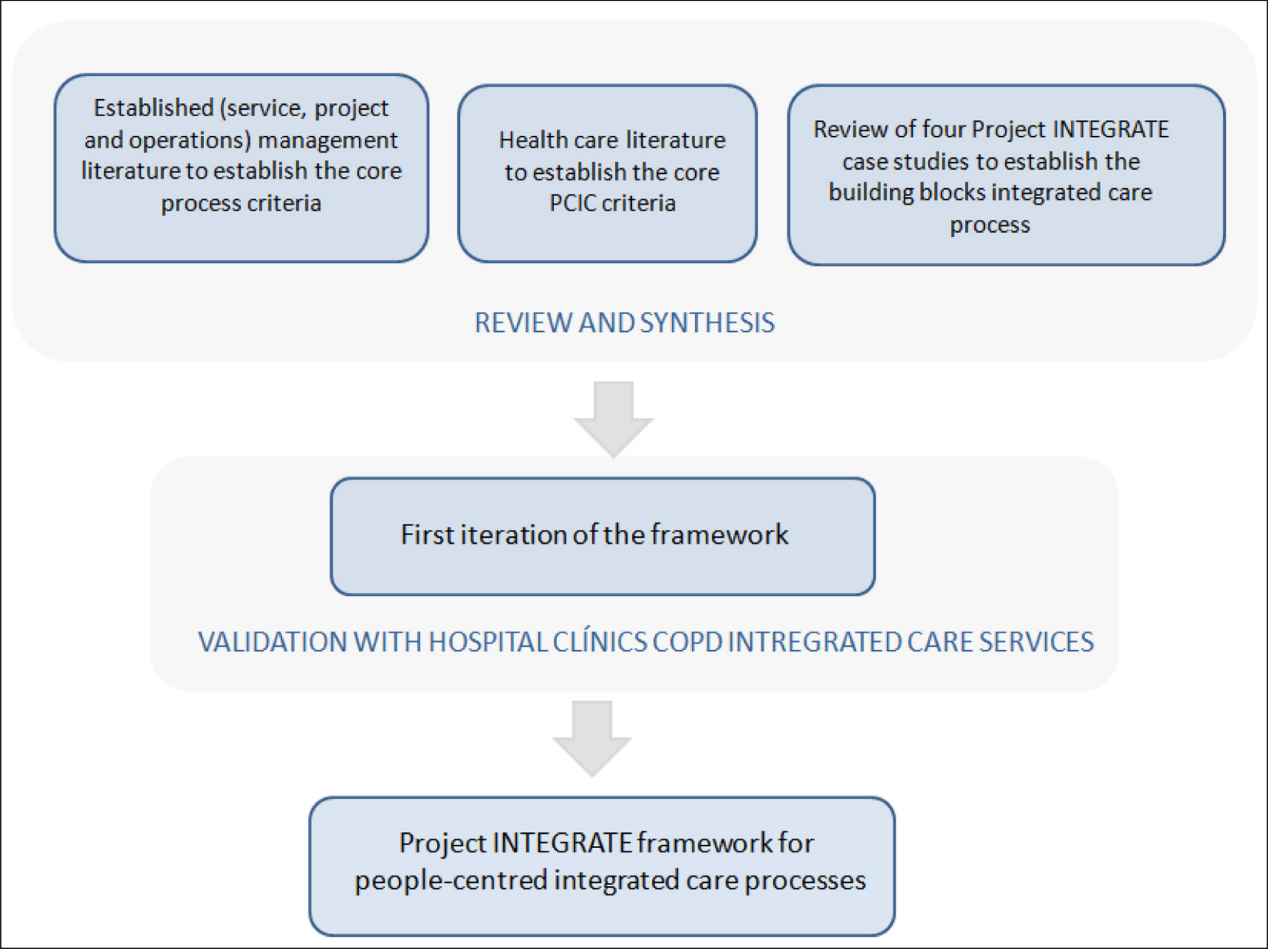
Development stages of the Project INTEGRATE people-centred integrated care process framework.

The framework was developed through a number of steps as follows (Figure 1).

Firstly, operations, service and project management literature were reviewed by the project team at IESE Business School in Barcelona, Spain to establish the core managerial and operational components of the framework that would be necessary to support sustainable, effective management and delivery of the PCIC process. The rationale behind such framework is that it should serve as a guide to managers to ensure every step of the process and service activity is analysed, and can be adapted in line with the reality of each service provider, and to help identify the key criteria for successful implementation of PCIC [35].

Conceptually speaking, activities taking place within a service provider can usually be classified into two distinct categories: processes and projects. A process can be defined as a sequence of interconnected activities with a repetitive nature and with an overall identifiable purpose [20]. However, in the case of the health care sector, and particularly with PCIC experiences which are developed with different levels of maturation, this distinction is less clear. Thus, pilot projects can be developed and coexist within larger processes of health service delivery. Therefore project management methodology can be extremely useful for managers in the design, implementation and evaluation of PCIC services. For example, to ensure a positive impact on all stakeholders, and with a primary focus on service-user satisfaction, firstly managers need to decide which project(s) should be carried out (in line with service users strategic objectives), and secondly, resources need to be organized and managed in such a way that the objectives/scopes of the selected project(s) can be reached within the given time and budget [36].

Furthermore, there are several important strategic questions to contemplate when planning and managing any type of project: what do we want to achieve, and why are we doing it?; how do we gauge its success?; what external conditions or factors must exist for the project to succeed?; and how are we going to do it? [36]. In addition, even though every project is different, a number of steps can always be commonly defined or classified within the process chain and should be identified from the offset within the project planning phase. Moreover, when examining capacities within a project or processes within a service provider, it is important to also assess the potential ‘waste’ i.e. any stage of the process that reduces the capacity of the entire process chain [36].

The European Foundation for Quality Management Excellence Model (EFQM) was selected as the basis of the framework, as it is an established self-assessment tool built on fundamental concepts and criteria that propose ways of achieving performance excellence, while accounting for different contexts and maintaining a focus on people. The model aims to assist to understand the strengths and potential performance gaps in relation to the service provider’s stated vision and mission by integrating current and planned activities to identify gaps and remove duplications, to provide coherent terminology to facilitate effective communication both within and outside service provider, as well as to provide a basic structure for management and monitoring to sustain results [37]. As such the model is a useful service improvement tool that facilitates the adoption of effective managerial practices into service delivery [38], and can also be used in conjunction with other managerial tools. There are a number of successful experiences of applying the EFQM approach in health care [31,38,39,40].

Secondly, the IESE-CRHIM Health Innovation InnPACT study and its Combined Innovation Project Charter Framework [41] were analysed and incorporated into our framework, as it includes a comprehensive evaluation framework that provides a standardized way to describe, evaluate and compare health innovation, accounting for all relevant stakeholders, and incorporates several other management concepts. The rationale is that the impact of innovation in health outcomes can be difficult to define and measure and usually surface in the medium term. The framework is applicable to innovations that are in different stages of completion, and aims to facilitate the analysis, comparison of initiatives and subsequent learning from this, as well as provide a set of attributes and criteria to measure the incremental value of a health care innovation [41]. The framework incorporates several other management concepts, it includes a self-assessment factsheet intended to be filled out by health care service-providers, as well as elements that assist to define the health care innovation based on the defined project life cycle [42]. Through the InnPACT study, the framework was applied and tested by several case studies within Spain to verify and demonstrate its practical utility.

Thirdly, a range of IESE teaching notes, and literature on process analysis and service design, operations and project management were reviewed [22,20,42,43,44], and a number of additional components/criteria were identified to include into our framework. For example, a process analysis work plan used in service design operations [42], as it elaborates a step-by-step process from a service-user perspective to gain a better understanding of the process, and to establish the potential impact of each activity performed can have on the service-user’s value perception (and satisfaction) of the service.

Furthermore, a review of the PCIC scientific and gray literature was conducted, to determine the criteria to develop and deliver PCIC, such as the types of human resources skill mix, the governance and financing structures, interoperable information communication technology requires, as well as how service users are involved in their own care plans [1,6,13,14,15,17,45,46,47,48,49]. To ensure coherence throughout the project, the same literature and methodological approach used to inform the development of the four Project INTEGRATE case studies [18,50,51] was used in the framework. However, some additional literature and criteria on people-centredness were included in line with the development of the integrated care field both within the scientific literature and policy discourse, to ensure the frameworks relevance beyond the life of the project.

At the same time, the project team in Fundacio Privada Clinic per a la Recerca Biomedica (FCRB)/Hospital Clínic, Barcelona, Spain, reviewed and compared the four Project INTEGRATE case studies to identify the generalizable building blocks of the integrated care process. The four case studies included two disease-pathways and two general conditions: Chronic Obstructive Pulmonary Disease (COPD) in Spain, diabetes in the Netherlands, geriatric condition in Germany and mental health care in Sweden [52,53]. The four case study reports were reviewed, relevant information was extracted to tabulate the care process narratives obtained from each case study; additional data were collected using summary tables that were sent out to the research teams based at the case study sites and any doubts were solved by direct contact.

Based on this review, the FCRB/Hospital Clínic project team established that the integrated care process can be organised into five main building blocks that take place chronologically as follows: 1) case identification/eligibility; 2) case evaluation; 3) definition of a work plan; 4) execution of the work plan, and 5) discharge/transition to another level/programme of care [54,55].

These findings were similar to those outlined in the work plan for process analysis in service design operation’s step-by-step process flow diagram [35,20] that aims to define the main stages of service process; the process flow diagram describes these main stages as: Access – Check in – Diagnostics – Delivery – Check-out – Follow-up. As such, this combined learning was incorporated into the framework by the IESE project team, and given that the target audience, the process flow diagram terminology was selected for use in the framework.

Lastly, this learning was synthesized to develop a first iteration of the framework. To determine and test whether it was a useful pragmatic tool that describes the essential steps of the PCIC process to be able to facilitate a comprehensive analysis of the service and that does not require special skills to apply, the framework was tested on a real integrated care case study analyzed under Project INTEGRATE, the Integrated Care Services for COPD patients at FCRB/Hospital Clínic which includes home hospitalisation and early discharge [56]. The Integrated Care Services programme leaders and hospitals managerial team that played a key role in the introduction of the programme into FCRB/Hospital Clínic, were asked via the FCRB/Hospital Clínic research team to apply the tool to their PCIC service and answer whether it was clear to understand, easy to apply and useful as an evaluation and improvement tool. Positive feedback was received, with some minor suggestions for improvement in terms of terminology used and clarity in some of the criteria instructions. This feedback was then incorporated into the framework by the IESE research team to create the final iteration of the framework.

## Results

Within each stage of the care process it is important to know what is happening, when and where, who is doing what, how it is done, and why – to understand what has prompted a given stage to occur. This understanding can then provide a rationale for the subsequent step in the care process chain. Furthermore, the rationale behind elaborating a step-by-step care process analysis, specifically from a service user perspective, is that stakeholders (particularly managers) can understand the care process and service better, in terms of establishing the value added that each activity performed can have on shaping the outcomes for service user as well as on the service provider. Thinking along these lines, can facilitate the ability to understand the needs and care preferences of the service user and to provide a service tailored to meet these needs – which is fundamental for the design and delivery of PCIC.

We present the PCIC process framework and detail each main section to better understand the criteria that facilitate integrated care processes. By using this framework, a multi-stakeholder approach can be taken in the evaluation of a PCIC process design and implementation. Similarly to the EFQM – which serves as a foundation for the framework – this is a self-assessment service improvement and best practice management tool that has been adapted specifically for PCIC. Table 1 details the PCIC process framework.

**Table 1:** Project INTEGRATE People Centred Integrated Care Process Framework.

The Project INTEGRATE framework to assess people-centred integrated care processes
**Strategy, Project Charter, Governance [37,41,46]:** This section includes a number of criteria to assist managerial teams to define how to organise, develop and facilitate the achievement of the mission and vision of a process or project; to create values required for long-term success and implement them via appropriate actions and behaviours; to support organisational policy and strategy; and to support the effective operation of its processes. This is compiled in a Project Charter – a blueprint that contains the vision and scope set out by leaders, and the roles and responsibilities of stakeholders, and a salient role for implementation. A Project Charter is a tangible reference point for focusing teams towards a common set of goals, which goes beyond describing the vision and responsibilities of stakeholders involved.
**Service name:** Add the title of the PCIC process being assessed or planned.
**Service description:** Describe the PCIC process being assessed or planned and what is included within it. What is the intended service innovation (i.e. the intended change)? Define the intended service users/target population.
**Mission:** Explain the rationale for the new PCIC process.
**Objectives:** Provide operational objectives that correspond to the mission statement. Are they specific; measurable; attainable; relevant and time-bound?
**Deliverables and milestones:** List all deliverables and milestones that will be achieved during this PCIC process (tangible and intangible).
**Scope:** State the boundaries of the PCIC process include what is included and required for the service delivery from your organisation, and what is required externally in order to successfully achieve this care process.
**Risk Management [20,41,42]:** In the design/planning phase, identify any assumptions or potential risks to the PCIC process and how this might impact the service user. This includes any aspects that can facilitate or complicate the development, and deployment of the care process and which focuses on the variability and the risks within the process, taking into account the impact these can have on the service provided. Attempt to classify the risk types identified ‘PESTEL analysis’ (i.e. political, economic, social, technological and legal) and these can be classified into the following broad service quality terms [20,41,42]: Reliability (e.g. coherence; consistency). Tangibles (e.g. facilities; personnel/staff; equipment and tools; documentation). Quality assurance (e.g. technical competence; professionalism; safety; confidentiality). Responsiveness (e.g. institutional response capacity and agility). Empathy (e.g. manner of communication). Actions for mitigating these risks should then be discussed in a suitable environment in which all the relevant stakeholders can be clear on how to minimise risk.
**Pre-defined ideal success criteria [41]:** Pre-define the ideal success criteria for the PCIC process, considering all stakeholders; note that this may or may not be the same as the objectives.
**Governance [37]:** Detail: 1) how to organize, develop and facilitate the achievement of the services mission and vision, and 2) how to create values required for long-term success, and 3) how to implement them via appropriate actions and behaviours.
**Degree and breadth of integration [13,14,48,49]:** Describe the current connectivity between the different PCIC process steps between and across different levels of services and other care providers and teams, degree and breadth of integration. Identify the essential elements that facilitate and/or hinder this. Within each of these stages it is important to know: when, where, and what is happening; who is doing what, and how it is done, including the supportive tools used. In addition to knowing why- as this can provide understanding on what prompted a given step of the process, and quite often provides a rationale for the subsequent step in the process chain.
**Leadership [37]:** Define the ‘leader(s)’ of this PCIC process. In this case, a leader is defined as being a role model striving for excellence and promoting a good communication strategy. Detail the people who lead the transitions between different stages of the case process, and who supports the integration of these care services.
**People [37]:** This section is about how an organisation coordinates, develops and releases knowledge, skills, and full potential of its people at an individual, group, and organisational levels. People centredness’ should underpin the entire care process design and therefore an assessment of how this is operationalised at each implementation stage is required.
**Professional Integration [13,14,42,47,48]:** Define the level of professional integration, in terms of joint working and group practices, contracting or strategic alliances of health professionals within and between institutions and organisation; an important distinction should be between whether these are ‘formal’ or ‘informal’ alliances. Define any person that will or could impact results of PCIC process; include any additional roles that have been created to support this innovation. Detail the multidisciplinary care plans and comprehensive assessment tools used by the team members. Detail whether the shared care protocols and procedures are in place and are well disseminated to the team(s); add a description of how the team may formally work together with such procedures and protocols. Finally, detail whether (and how much) investment is demanded by understanding the needs of providers and team. Define the additional skill sets and/or education required for the service providers and multidisciplinary team members involved in the PCIC process in order to fulfil their ‘new roles’ to support PCIC (in comparison to ‘traditional care’). Define any new task delegation or advanced roles that may be required to support this process innovation (e.g. from doctors to nurse) [57].
**People centredness [58,26]:** At what stages of the integrated care process are service users involved in their decision-making? How are they involved (i.e. what type of self-management support and/or support for informal carers offered)? How are service user needs and their feedback incorporated into the evaluation and design of the service in order to co-design solutions? If this is not done, discuss how this might be done. Identify the skill sets and education required for service users to be able to be involved in the PCIC process (e.g. health literacy)?
**Resources [37]:** This section relates to the enablers or barriers to PCIC and therefore the necessary internal resources required in order to support effective operation. E.g. information management, financial incentives and reimbursement structures, facilities and infrastructure and human resources. In addition, how an organisation plans and manages its external partnerships and internal resources in order to support its policy and strategy and the effective operation of its processes.
**Information management:** Detail the current information system(s) used to facilitate the PCIC process and their level of interoperability (e.g. shared electronic medical records, risk stratifications). What services do they connect? What services do they still need to connect?
**Finance:** Detail the financial incentives and reimbursement structure that are used and/or that can be used to facilitate the PCIC process. Include how these are different from standard care.
**Facilities and infrastructure:** Detail the necessary facilities (space, equipment, materials and technological support) to enhance the PCIC process. What additional infrastructure is required compared to standard care?
**Care process [37]:** This section assists to outline and clearly define the building blocks of the PCIC process, and the transition from one block to another, which typically involves a mix of situations (e.g. responsibility handover, completion-start of tasks, information transfer), and the identification of the value propositions and how to generate increased value for all stakeholders [46].
**Building blocks of integrated care process [20]:** Define each step of the care process, adding details about which of these steps (and which parts of these steps) are ‘visible to’ or ‘actively experienced by’ the patient and what is not but is necessary to support the care process, in terms of: access; check-in; diagnostics; delivery; check out/follow up: **Access:** Define how the ‘case identification’ process and ‘service user admissions’ are organised and performed. **Check-in:** Detail how the ‘case evaluation’, ‘service user assessment and enrolment’ of the service user into the PCIC service are conducted. **Diagnostics:** Explain the ‘work plan definition’ and ‘care plan development’ processes. **Delivery:** Detail how the ‘work plan execution and ‘treatment’ are performed and defined. **Check out/follow up:** Describe the ‘service user’s transition/discharge’ process and the organisation of subsequent services and visits. How are service users being supported to self-manage their care?
**Identification of value proposition and waste [22,41,42,20,44]:** Define the essential promoter(s) and potential inhibitors of the ‘integratedness’ of the care process. Analyse the stages of the care process and the components, while considering what value they bring to the different stakeholders. These stages can be optimised – in light of a needs analysis – or alternatively removed. High-value added and urgent changes should be prioritised to leave a larger positive impact. Short-term gains provide direction; motivate health and social care workers and aid in scaling and transferring practices. Furthermore, compare the PCIC with standard care, in terms of the value proposition and the value added to stakeholders. Assess whether an activity or criteria within a process is adding value or not using the following categories; if it does not contribute to either value potential or realization, it should be removed [44]: **Value-Added Activities** – an activity that is transformative if the service user needs and prefers it. **Needed or Enabling Activity** – if no value is created with this activity, but the activity cannot be eliminated as it is a necessary part of the current process. **Non Value-Added Activity** – if the activity consumes resources but does not create value to the service user and can be removed without hindering the process then it is considered as ‘waste’ and should be removed.
**Results/monitoring and evaluation of impact [37]:** This section assists to assess impact of the care process innovation on stakeholders. In addition to a-posteriori assessment of the entire integrated care process [37,47,48,49,42].
**Service user results:** What impact has the PCIC process/had on the service user(s) (i.e. how successful was the innovation)? What was the perceived impact and value added by the service users? [42] How this is measured? How often this is measured? How is the feedback incorporated?
**Health professional results:** What impact has the PCIC process had on the health professionals within the service provider(s)? What was the perceived impact and value added for the professionals involved? [42] How often is this measured and how? How is this feedback incorporated and used?
**Service provider results:** What impact has the PCIC process/service innovation had on the service provider? Has there been a change in the utilization of services and in expenditure since the PCIC process/service innovation? What was the perceived impact and value added by the service provider? [42] How often is this measured? How is this measured? How is this feedback incorporated/used?
**Care process design review:** Finally, review the assessment of the current PCIC process, compare it with the pre-defined ideal success criteria and then try to summarize the main changes, value-added and/or ‘waste’ identified. From this comparison, the care process can then be optimised to ensure service quality and effectiveness that will support potential scale-up and transferability to other settings [20].

## Discussion

There is a significant knowledge gap in terms of supporting the ‘how to’ implement PCIC and this framework, developed under the European Commission’s FP7 funded Project INTEGRATE, aims to be a pragmatic addition to the tools already available to integrated care managers. In this paper we present this framework that aims to provide practical guidance for managers and planners with the design, implement and evaluate PCIC services of all levels of maturation. Managers and planners can use the tool to conduct a step-by-step self-assessment of the PCIC process and the steps and criteria involved, as well as the value added to stakeholders at the different implementation stages, to gain insights on how to ensure effective PCIC service delivery.

Overall, this tool was built from the synthesis of lessons learnt and best practices from the PCIC literature and the operations, service and project management research fields, as well as real integrated care case studies, and therefore represents a novel and comprehensive approach to support PCIC process design, implementation and evaluation.

To begin, the framework was built around EFQM [37] that propose ways of achieving excellence and quality performance, while accounting for different contexts and maintaining a focus on people, therefore making it suitable for analyzing PCIC in different settings and for different service user groups. Whilst the framework appears to present the PCIC process as static/linear process, this of course is not the case in practice and the tool can be applied to assess the degree and breadth of the integration of services along the different stages of implementation.

Furthermore, the tool combines a number of important best practice considerations and criteria that have not been included in previous integrated care assessment frameworks. For example, the inclusion of the pre-defined ideal success criteria which assists to pre-define what success would mean for the specific PCIC service, in the context of the planned service innovation and in line with the initial aims and objectives. In addition, the inclusion of a risk assessment assists to anticipate risks, adverse events and possible ‘waste’ within the process (i.e. anything that does not add value to stakeholders.

Moreover, the identification of value proposition to stakeholders also supports the distinction between perceived and real value-added to stakeholders at each stage of the PCIC process compared to standard care, and assists to identify where to remove any potential ‘waste’ from the process. Determining and identifying value proposition (i.e. the value added by an activity) involved in a process chain is useful to determine whether a particular entity is/should be included, in terms of the value it is adding and whether the value pertains to satisfying needs. This can also assist to better understand the preserved value and how to enhance value in line with the resources available to improve the PCIC process capacity [22,41,44]. Value and quality are related, but independent concepts; quality is thought to be the alignment of the service provider performance with service users expectations, as well as with service user needs [44] – which are crucial aspects of effective PCIC services.

To establish the main integrate care process building blocks, four integrated care case studies from across Europe were analyzed. These cases were initially selected for study under Project INTEGRATE as they were good practices/early implementers of integrated care across Europe addressing several conditions of high epidemiologic importance and high economic burden on health systems, implemented in different types of health systems: national health systems (Spain and Sweden) and health insurance (Netherlands and Germany) (for further details refer to [18,51]).

Once a first iteration of the framework was established, the FCRB/Hospital Clínic COPD integrated care case study was used to test the specificity of the framework. However, in terms of limitations, the framework has not been externally verified by all Integrated Care Unit staff at FCRB/Hospital Clínic or in all of the programmes. In addition, as the tools sensitivity has only been tested in one case study, and given the high degree of heterogeneity of PCIC programs in place and envisioned, further validation of the tool is needed, for example in nascent PCIC services and in settings beyond those of the Project INTEGRATE case studies.

Future work should therefore consider testing and applying this framework in diverse integrated care settings. Moreover, there is still a need to support managers on how to adopt ‘successful’ PCIC experiences from other settings into their own context, all to effectively guide the orientation of the model of care along the different avenues of integration [5,59,60].

This PCIC process framework fed into the final phase of Project INTEGRATE, which was to elaborate practical recommendations and managerial lessons, as well as an Integrated Care Management Excellence Framework [61,62]. The aim of the Management Excellence Framework is to provide an operational perspective for improving processes and resource management within the boundaries of integrated care, and an accompanying toolkit was developed to assist health care leaders to assess the performance of their organisations, identify areas of deficiencies and to construct solutions in a collaborative manner. This work was also complimentary to other on-going work initiated under Project INTEGRATE that aims to develop a comprehensive framework for implementing integrated care [63].

## Conclusion

People-centred integrated care is a widely acknowledged approach to improve the quality and effectiveness of health and social care services. Establishing effective people-centred integrated care services can lead to improvements in the quality and performance of health and social care for people living with chronic and complex conditions, and can ensure that they receive the necessary care in line with their particular needs and preferences. The development and use of evidence-based guidelines and tools are useful to support the establishment of these types of service innovations. Nevertheless, there is still a need for in-depth understanding of how to and effectively design, implement and evaluate of these service innovations, as well as how to transfer successful experiences to other settings.

Under the European Commission’s FP7 funded Project INTEGRATE, a framework was developed to provide pragmatic guidance to managers and planners on the design, implementation and evaluation phases of effective people-centred integrated care service delivery. The framework was developed through a series of steps: a review and synthesis of relevant operations, project and managerial literature, as well as health care literature, and an analysis of four good practice integrated care case studies based across Europe. The first interaction of the framework was then applied to one of the established integrated care case studies to test and validate its relevance and its ease of use by health care professionals before establishing a final version.

The framework includes a number of important considerations and criteria not previously included in integrated care assessment frameworks e.g. the inclusion of the pre-defined ideal success criteria which assists to pre-define what a successful outcome would mean in line with the initial aims and objectives, a risk assessment, and the identification of potentially wasteful activities within the process, as well as the identification of value proposition to distinguish between perceived and real value-added to stakeholders at each stage of the people-centred integrated care process compared to standard care.

This framework has the potential to support managers to effectively design, implement and evaluate people-centred integrated care processes; further application of this framework to other setting external to Project INTEGRATE is warranted to establish its utility as either a stand-alone tool or as a complementary tool that can be combined with other tools. Such tools can be used to support comprehensive people-centred integrated care performance and service delivery and provide a range of benefits to service users in line with their health needs and care preferences.

## References

[1] Stein, VK, Barbazza, ES, Tello, J and Kluge, H. Towards people-centred health services delivery: a Framework for Action for the World Health Organisation (WHO) *European Region* Int J Integr Care. 2013; 13: e058 Ubiquity Press Available: http://www.ncbi.nlm.nih.gov/pubmed/24409110. 24409110 10.5334/ijic.1514 PMC3886596

[2] Goodwin, N. Towards People-Centred Integrated Care: From Passive Recognition to Active Co-production? Int J Integr Care. 2016; 16 Ubiquity Press DOI: 10.5334/ijic.2492 PMC5015546 27616970

[3] Boulding, W, Glickman, SW, Manary, MP, Schulman, KA and Staelin, R. Relationship between patient satisfaction with inpatient care and hospital readmission within 30 days. Am J Manag Care, 2011; 17: 41–8. Available: http://www.ncbi.nlm.nih.gov/pubmed/21348567. 21348567

[4] World Health Organization. Everybody’s business: strengthening health systems to improve health outcomes: WHO’s framework for action [Internet] World Health Organization; 2007 Available: https://www.scribd.com/document/123877628/Everybody-s-Business-Strengthening-Health-Systems-to-Improve-Health-Outcomes.

[5] Cash-Gibson, L, Tello, J, Allen, L and Toro, N. Integrating health services: Policy brief Technical Series on Primary Health Care. World Health Organization [Internet]; 2018 Available: http://www.who.int/docs/default-source/primary-health-care-conference/linkages.pdf?sfvrsn=bfbb4059_2.

[6] World Health Organization. Global strategy on people-centred and integrated health services [Internet] Geneva; 2015 Available: http://www.who.int/servicedeliverysafety/areas/people-centred-care/global-strategy/en/.

[7] World Health Organization. The world health report 2000 - Health systems: improving performance [Internet] Geneva: World Health Organization; 2000 Available: http://www.who.int/whr/2000/en/.

[8] Tello, J and Barbazza, E. Health services delivery: A concept note (2015) [Internet] Copenhagen; 2015 Available: http://www.euro.who.int/en/health-topics/Health-systems/health-services-delivery/publications/2015/health-services-delivery-a-concept-note-2015.

[9] World Health Organization. The European Framework for Action on Integrated Health Services Delivery [Internet] Cophenhagen: World Health Organization, Regional Office for Europe; 2016 Available: http://www.euro.who.int/en/health-topics/Health-systems/pages/publications/2016/eurrc6615-strengthening-people-centred-health-systems-in-the-who-european-region-framework-for-action-on-integrated-health-services-delivery.

[10] World Health Organization. Framework on integrated people-centred health services. Report by the Secretariat. World Health Organization. Sixty-ninth World Health Assembly. WHA69.39 Geneva; 2016.

[11] Hanefeld, J, Powell-Jackson, T and Balabanova, D. Understanding and measuring quality of care: Dealing with complexity. Bull World Heal Organ; 2017 DOI: 10.2471/BLT.16.179309 PMC5418826 28479638

[12] Minkman, MMN. Values and Principles of Integrated Care Int J Integr Care, 2016; 16 Ubiquity Press DOI: 10.5334/ijic.2458 PMC5015537 27616947

[13] Goodwin, N. Understanding Integrated Care Int J Integr Care, 2016; 16 Ubiquity Press DOI: 10.5334/ijic.2530 PMC5354214 28316546

[14] Valentijn, PP, Schepman, SM, Opheij, W and Bruijnzeels, MA. Understanding integrated care: A comprehensive conceptual framework based on the integrative functions of primary care Int J Integr Care, 2013; 13 Ubiquity Press DOI: 10.5334/ijic.886 PMC3653278 23687482

[15] World Health Organization. People-centred and integrated health services: An overview of the evidence [Internet] Geneva; 2015 Available: http://www.who.int/servicedeliverysafety/areas/people-centred-care/evidence-overview/en/.

[16] Sheikh, K, Ranson, MK and Gilson, L. Explorations on people centredness in health systems Health Policy Plan, 2014; 29(Suppl 2): ii1–5. Oxford University Press DOI: 10.1093/heapol/czu082 25274634 PMC4202918

[17] Martínez-González, NA, Berchtold, P, Ullman, K, Busato, A and Egger, M. Integrated care programmes for adults with chronic conditions: A meta-review. Int J Qual Heal Care, 2014; 26: 561–570. DOI: 10.1093/intqhc/mzu071 PMC4195469 25108537

[18] Cash-Gibson, L and Rosenmoller, M. Project INTEGRATE - a common methodological approach to understand integrated health care in Europe Int J Integr Care, 2014; 14 Ubiquity Press DOI: 10.5334/ijic.1980 PMC4276036 25550690

[19] de Bruin, SR, Stoop, A, Billings, J, Leichsenring, K, Ruppe, G, Tram, N, et al. The SUSTAIN Project: A European Study on Improving Integrated Care for Older People Living at Home Int J Integr Care. 2018; 18 Ubiquity Press DOI: 10.5334/ijic.3090 PMC5887072 29632456

[20] Ribera, J. Process Analysis in Service Delivery Operations. Workbook [Internet]; 2006 Available: http://www.iesep.com/es/process-analysis-in-service-delivery-operations-workbook-9731.

[21] Goodwin, N. How do you build programmes of integrated care? The need to broaden our conceptual and empirical understanding Int J Integr Care, 2013; 13: e040 Ubiquity Press Available: http://www.ncbi.nlm.nih.gov/pubmed/24179459. 24179459 10.5334/ijic.1207 PMC3812348

[22] Moscoso, P and Ribera, J. A Project Management Methodology [Internet]; 2013 Available: http://www.iesep.com/es/a-project-management-methodology-89184.

[23] Van Houdt, S, Heyrman, J, Vanhaecht, K, Sermeus, W and De Lepeleire, J. An in-depth analysis of theoretical frameworks for the study of care coordination Int J Integr Care. 2013; 13 Ubiquity Press DOI: 10.5334/ijic.1068 PMC3718267 23882171

[24] Busetto, L, Luijkx, K, Huizing, A and Vrijhoef, B. Implementation of integrated care for diabetes mellitus type 2 by two Dutch care groups: A case study BMC Fam Pract., 2015; 16: 105 BioMed Central DOI: 10.1186/s12875-015-0320-z 26292703 PMC4546228

[25] Barr, V, Robinson, S, Marin-Link, B, Underhill, L, Dotts, A, Ravensdale, D, et al. The Expanded Chronic Care Model: An Integration of Concepts and Strategies from Population Health Promotion and the Chronic Care Model. Healthc Q, 2003; 7: 73–82. DOI: 10.12927/hcq.2003.16763 14674182

[26] Kaehne, A. The Building Blocks of Integrated Care Int J Integr Care, 2016; 16 Ubiquity Press DOI: 10.5334/ijic.2527 PMC5354202 28316545

[27] Proctor, EK, Powell, BJ and McMillen, JC. Implementation strategies: Recommendations for specifying and reporting. Implement Sci. BioMed Central; 2013; 8: 139 DOI: 10.1186/1748-5908-8-139 PMC3882890 24289295

[28] Womack, JP and Jones, DT. Lean thinking banish waste and create wealth in your corporation Free Press; 2003.

[29] Kenney, C. Transforming health care: Virginia Mason Medical Center’s pursuit of the perfect patient experience CRC Press; 2011.

[30] Bohmer, RMJ and Ferlins, E. Virginia Mason Medical Center Harvard Business School Case 606-044 [Internet]. Boston, MA: Harvard Business School Publishing; 2005 Available: https://www.hbs.edu/faculty/Pages/item.aspx?num=32725.

[31] Minkman, M. The Development Model for Integrated Care: A validated tool for evaluation and development. J Integr Care, 2016; 24: 38–52. DOI: 10.1108/JICA-01-2016-0005

[32] Goodwin, N, Stein, V and Amelung, V. What Is Integrated Care? As part of Cham: Springer International Publishing., 2017; 3–23. DOI: 10.1007/978-3-319-56103-5_1

[33] Project INTEGRATE website [Internet]. Available: http://www.projectintegrate.eu.com.

[34] Borgermans, L, Marchal, Y, Busetto, L, Kalseth, J, Kasteng, F, Suija, K, et al. How to Improve Integrated Care for People with Chronic Conditions: Key Findings from EU FP-7 Project INTEGRATE and Beyond Int J Integr Care. 2017; 17 Ubiquity Press DOI: 10.5334/ijic.3096 PMC5854097 29588630

[35] Munoz-Seca, B. Technical Note: A Model for Configuring operations in Service Companies. PN-495-E; 2014.

[36] Moscoso, PG and Ribera, J. Technical note: A project Management Methodology. PN-493-E; 2013.

[37] European Foundation for Quality Management. An Overview of the EFQM Excellence Model [Internet]; 2013 Available: http://www.efqm.org/sites/default/files/overview_efqm_2013_v1.1.pdf.

[38] Favaretti, C, De Pieri, P, Torri, E, Guarrera, G, Fontana, F, Debiasi, F, et al. An EFQM excellence model for integrated health care governance. Int J Health Care Qual Assur., 2015; 28: 156–172. DOI: 10.1108/IJHCQA-02-2014-0022 26335168

[39] Moeller, J. The EFQM Excellence Model. German experiences with the EFQM approach in health care. Int J Qual Heal care J Int Soc Qual Heal Care, 2001; 13: 45–9. Available: http://www.ncbi.nlm.nih.gov/pubmed/11330442. 10.1093/intqhc/13.1.45 11330442

[40] Rosenmöller, M. Quality in Health Management, The European Quality Model applied to the Health Sector. Experience from Catalonia. The 1996 Quality Conference in Europe, The Conference Board and the (EFQM) Berlin; 1996.

[41] Ribera, J, Rosenmöller, M and Borrás, P. Health care Innovation Impact Study: InnPACT [Internet]; 2013 Available: http://www.iese.edu/research/pdfs/ST-0271-E.pdf.

[42] Ribera, J. The Project Life Cycle: Definition [Internet]; 2011 Available: http://www.iesep.com/es/the-project-life-cycle-definition-11156.

[43] Muñoz-Seca, B. A Model for Configuring Operations in Service Companies [Internet]; 2014 Available: http://www.iesep.com/es/a-model-for-configuring-operations-in-service-companies-97579.html.

[44] Sampson, SE. Essentials of service design and innovation: Third edition Brigham Young University; 2014.

[45] Ferrer, L and Goodwin, N. What are the principles that underpin integrated care? Int J Integr Care. 2014; 14 Ubiquity Press DOI: 10.5334/ijic.1884 PMC4251472 25473383

[46] Suter, E, Oelke, ND, Adair, CE and Armitage, GD. Ten key principles for successful health systems integration. Healthc Q. PMC Canada manuscript submission, 2009; 13(Spec No): 16–23. Available: http://www.ncbi.nlm.nih.gov/pubmed/20057244. 10.12927/hcq.2009.21092 PMC3004930 20057244

[47] Goodwin, N. Thinking differently about integration: people-centred care and the role of local communities Int J Integr Care. 2014; 14: e026 Ubiquity Press Available: http://www.ncbi.nlm.nih.gov/pubmed/25337063 25337063 10.5334/ijic.1736 PMC4203115

[48] Kodner, DL. All together now: A conceptual exploration of integrated care. Healthc Q., 2009; 13(Spec No): 6–15. Available: http://www.ncbi.nlm.nih.gov/pubmed/20057243 DOI: 10.12927/hcq.2009.21091 20057243

[49] Goodwin, N, Sonola, L, Thiel, V and Kodner, DL. Co-ordinated care of people with complex chronic conditions: Key lessons and markers for success [Internet]. Available: https://www.kingsfund.org.uk/publications/co-ordinated-care-people-complex-chronic-conditions.

[50] Kodner, DL and Spreeuwenberg, C. Integrated care: Meaning, logic, applications, and implications - a discussion paper Int J Integr Care. 2002; 2 Ubiquity Press DOI: 10.5334/ijic.67 PMC1480401 16896389

[51] Cash-Gibson, L and Rosenmoller, M. Rationale for project INTEGRATE [Internet]; 2013 Available: https://integratedcarefoundation.org/blog/rationale-for-project-integrate.

[52] Busetto, L. Great Expectations: The Implementation of Integrated Care and Its Contribution to Improved Outcomes for People with Chronic Conditions Int J Integr Care. 2016; 16 Ubiquity Press DOI: 10.5334/ijic.2555 PMC5354216 28316556

[53] Case Studies - Project INTEGRATE [Internet]. [cited 28 1 2018]. Available: http://www.projectintegrate.eu.com/case-studies.

[54] Alonso, A and Kubesch, N. Project INTEGRATE: Care Process Design: A fundamental piece or a superfluos effort? Fundació Clínic per a la Recerca Biomèdica (FCRB). The 15th International Conference on Integrated Care Edinburgh; 2015 Available: http://www.projectintegrate.eu.com/wp-content/uploads/2015/05/3.5-Alonso_Albert-Care-Process-Design.pdf.

[55] Alonso, A, Kubesch, N, Sampietro-Colom, L, Cash-Gibson, L, Tigova, O, Ribeiro, M, et al. Project INTEGRATE: Deliverable D6.1 Final Report on Care Process Design [Internet]. 2015 Available: www.projectintegrate.eu.

[56] Project INTEGRATE - COPD -Integrated Care Case Study. [Internet]. 2015 [cited 28 1 2018]. Available: http://www.projectintegrate.eu.com/integrated-care/research/phase1-case-studies/copd.

[57] Dussault, G and Buchan, J. Chapter 11: Noncommunicable diseases and human resources for health: A workforce fit for purpose Part of Health systems respond to noncommunicable diseases: Time for ambition. [Internet]. Jakab, M, Farrington, J, Borgermans, L and Mantingh, F (eds.). World Health Organization Regional Office for Europe; 2018 Available: http://www.euro.who.int/en/publications/abstracts/health-systems-respond-to-noncommunicable-diseases-time-for-ambition-2018.

[58] Borgemans, L and Nolte, E. Chapter 10: A people-centred approach to strengthening health systems for noncommunicable diseases Part of Health systems respond to noncommunicable diseases: Time for ambition. [Internet]. Jakab, M, Farrington, J, Borgermans, L and Mantingh, F (eds.). Copehagen: World Health Organization Regional Office for Europe; 2018 Available: http://www.euro.who.int/en/publications/abstracts/health-systems-respond-to-noncommunicable-diseases-time-for-ambition-2018.

[59] Support you can expect from WHO for IPHCS [Internet]. World Health Organization; 2018 Available: http://www.who.int/servicedeliverysafety/areas/people-centred-care/support/en/.

[60] EFFA IHSD implementation package [Internet]. World Health Organization Regional Office for Europe. Available: http://www.euro.who.int/en/health-topics/Health-systems/health-services-delivery/european-framework-for-action-on-integrated-health-services-delivery-effa-ihsd.

[61] Binkley, G, Olena, T, Kubesch, N, Kieselev, J, Alonso, A, Ribera, J, et al. Project INTEGRATE: D12.1 Report: Managerial lessons learnt [Internet]. 2016 Available: www.projectintegrate.eu.

[62] Binkley, G, Tigova, O, Cash-Gibson, L, Alonso, A and Rosenmöller, M. Leading Change towards Integrated Care: Lessons from Hospital Clínic Barcelona. Part of Designing Integrated Care Ecosystems: A Socio-Technical Perspective (Book chapter, book publication pending).

[63] González-Ortiz, LG, Calciolari, S, Goodwin, N and Stein, V. The Core Dimensions of Integrated Care: A Literature Review to Support the Development of a Comprehensive Framework for Implementing Integrated Care Int J Integr Care. 2018; 18 Ubiquity Press DOI: 10.5334/ijic.4198 PMC6137610 30220893

